# Longitudinal strain bull’s eye plot patterns in patients with cardiomyopathy and concentric left ventricular hypertrophy

**DOI:** 10.1186/s40001-016-0216-y

**Published:** 2016-05-10

**Authors:** Dan Liu, Kai Hu, Peter Nordbeck, Georg Ertl, Stefan Störk, Frank Weidemann

**Affiliations:** Comprehensive Heart Failure Center, Würzburg, Germany; Department of Internal Medicine I, University Hospital Würzburg, Würzburg, Germany; Innere Klinik II, Medical Clinic II, Katharinen-Hospital, Obere Husemannstraße 2, 59423 Unna, Germany

**Keywords:** Speckle tracking imaging, Bull’s eye plot, Cardiomyopathy, Left ventricular hypertrophy

## Abstract

Despite substantial advances in the imaging techniques and pathophysiological understanding over the last decades, identification of the underlying causes of left ventricular hypertrophy by means of echocardiographic examination remains a challenge in current clinical practice. The longitudinal strain bull’s eye plot derived from 2D speckle tracking imaging offers an intuitive visual overview of the global and regional left ventricular myocardial function in a single diagram. The bull’s eye mapping is clinically feasible and the plot patterns could provide clues to the etiology of cardiomyopathies. The present review summarizes the longitudinal strain, bull’s eye plot features in patients with various cardiomyopathies and concentric left ventricular hypertrophy and the bull’s eye plot features might serve as one of the cardiac workup steps on evaluating patients with left ventricular hypertrophy.

## Background

Left ventricular hypertrophy (LVH) is a common imaging finding in daily clinical practice. LVH can be detected in athletes following long-term exercise training, in hypertensive and aortic stenosis patients due to persistent pressure overload, in hypertrophic cardiomyopathy patients, and in patients with systemic diseases such as amyloidosis, Fabry disease, Friedreich’s ataxia. Echocardiography plays an important role on detecting LVH and underlying causes in current clinical practice [1, 2]. Nowadays, speckle tracking imaging (STI) technique is used to quantify global and regional myocardial deformation [3]. Its clinical application has been intensively studied in patients with various cardiovascular disorders over the last decade [4]. STI-based automated function imaging (AFI) is a user friendly advancement to evaluate left ventricular (LV) systolic function and regional patterns based on regional LV longitudinal strain values [5]. The result of AFI is usually presented as a bull’s eye plot showing color-coded and numerical values for peak systolic longitudinal strain of all LV segments. This bull’s eye plot provides an intuitive overview of LV systolic function status in a single diagram. A comprehensive demonstration on the typical bull’s eye plot patterns of various cardiomyopathy patients with LVH in the literature is sparse. In the present review, we summarized the features and clinical application of bull’s eye plots in patients with various cardiomyopathies and concentric LVH.

## Review

### Longitudinal strain bull’s eye plot acquisition

The bull’s eye plot can be acquired either by AFI algorithm or standard two-dimensional (2D) strain algorithm. Both methods are based on 2D STI, with quantitative information generated by measuring longitudinal strain from three apical views (apical long-axis view, 4- and 2-chamber views) with frame rates between 50 and 80 frames per second.

AFI is performed on apical views in the following order: apical long-axis, 4-chamber and 2-chamber view. A region of interest (ROI) is defined by a three-point click method, with two points placed on each side of the mitral annulus and a third point at the apex, followed by automated tracing of endocardial and epicardial borders. After validation of the tracking quality, aortic valve closure timing has to be defined. It is usually done by defining the end of the T-wave of the corresponding electrocardiographic tracing [6].

To obtain the bull’s eye plot with the standard 2D strain method, the timing of aortic valve closure should be first determined using continuous-wave Doppler across the aortic valve in apical 5-chamber view. Then, the ROI is created by manually applying successive points along the endocardial border in the three apical views at end-systolic frame [6].

For both methods, the system automatically tracks the tissue within the ROI throughout the cardiac cycle. The LV is divided into six segments in each apical view, accounting for a total of 18 segments covering the entire LV from base to apex [7]. After validation of the automatic tracking, the ROI can be manually adjusted for each segment if necessary to ensure the best tracking quality. Most of processing workstations offer an automated evaluation of tracking quality. In our experience, the tracking quality evaluation done automatically by system is not always correct. Thus, the tracking for each segment must be visually controlled and validated by the operator. If necessary, the operator should check the tracking from frame to frame. Correct ROI definition is crucial to get good tracking. Incorrect placement of the basal or apical points when defining ROI, as well as too narrow or too wide ROI width are the common reasons for bad tracking. Noise or reverberations due to image quality is also one reason for failing to tracking. In addition, technical attention is required in some Fabry patients and hypertensive patients with a prominent septal bulge. Because some end-stage of Fabry patients may present an asymmetric LV remodeling and lateral wall became thinner than the septal wall, the ROI at the lateral wall should therefore be adjusted to match the thinner wall thickness. However, the ROI should be “enlarged” to match the prominent septal bulge of hypertensive patients.

**Fig. 1:**
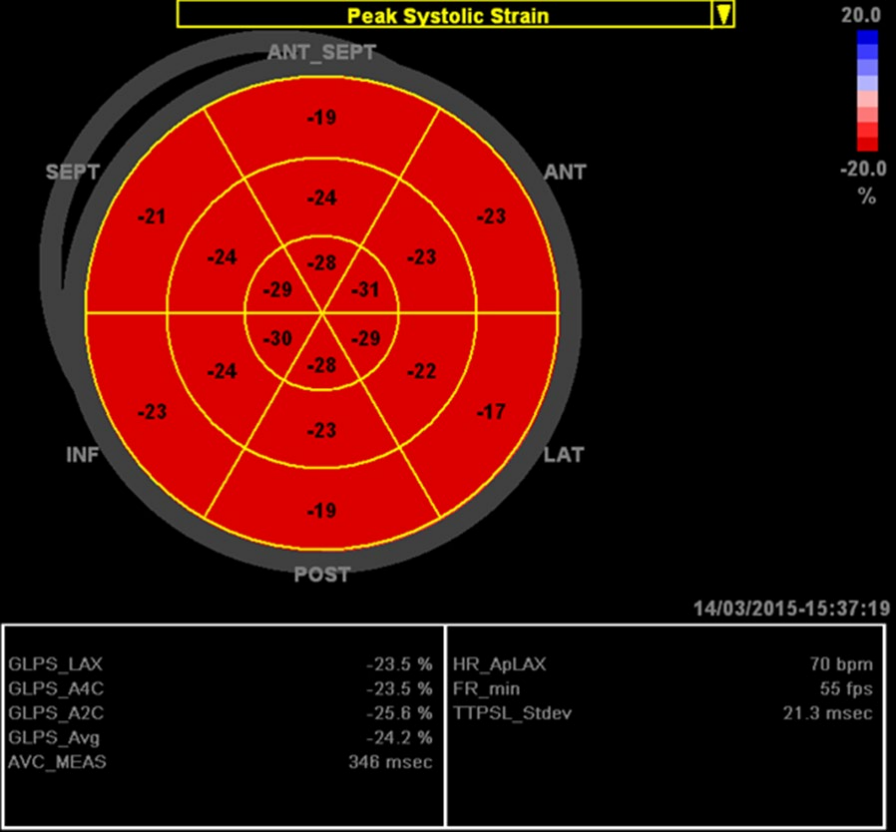
Example of the longitudinal strain bull’s eye plot derived from two-dimensional speckle tracking imaging in a healthy subject (50-year-old female)

Peak systolic longitudinal strain for each segment, global strain for each view, and average strain for the whole LV can be derived from both methods. The bull’s eye plot can be configured to display either 18 or 17 segments. The magnitude and homogeneity of longitudinal strain for each segment are displayed in an intuitively color-coded polar map (red–pink–blue), where the inner ring represents the apex of the LV, the middle ring represents the mid segments and the outer ring represents the basal segments. Bright red denotes normal strain values (<−16 %), light red denotes reduced value (−16 to −11 %), light pink (−10 to −6 %) and pale pink (−5 to 0 %) denotes severely reduced values, and blue denotes a positive value suggesting paradoxical systolic expansion. In a healthy subject, uniformly red pattern of the bull’s eye plot represents a normal range in strain values (i.e., varying from −16 to −22 %, Fig. 1) [8].

Currently, there are several commercial post-processing software products available for speckle tracking imaging analysis and longitudinal strain bull’s eye plot, including EchoPAC Healthcare from GE device and QLAB (cardiac motion quantification, CMQ) from Philips Medical Systems etc. The bull’s eye plots displayed in this review are generated using EchoPAC software by GE Vivid E9. Previous studies showed that the values of global longitudinal systolic strain obtained with Philips and GE echo systems have good correlations either in healthy population or patients [9–11]. The better agreement is observed in cardiac patients than in healthy controls [11]. A meta-analysis on global systolic strain in normal adults indicated that the values measured by the EchoPAC software were similar as the values by non-EchoPAC software (19.65 ± 1.78 vs. 19.67 ± 1.80 %) [12]. A recent report showed that global longitudinal systolic strain obtained from Philips and GE echo stations was comparable but the strain values of the basal segment obtained from Philips and GE echo stations were not comparable; thus, caution is needed on interpreting the bull's eye patterns from the two venders [11].

### Longitudinal strain bull’s eye plot patterns in patients with hypertrophic cardiomyopathies

#### Athlete’s heart

Physiological hypertrophy can be detected in athletes’ heart. Slightly enlarged left cardiac chamber, increased LV mass, and modestly enlarged aortic root can be visualized on conventional echocardiography. LVH in these subjects is generally symmetric and LV septal thickness is usually <13 mm in men and <11 mm in women [13]. LV ejection fraction (EF) and diastolic function remain normal. Some athletes may present enhanced early diastolic LV filling [14]. Assessment of diastolic function might thus be a key factor for differentiating physiological LVH due to exercise adoption from pathological LVH [15].

**Fig. 2:**
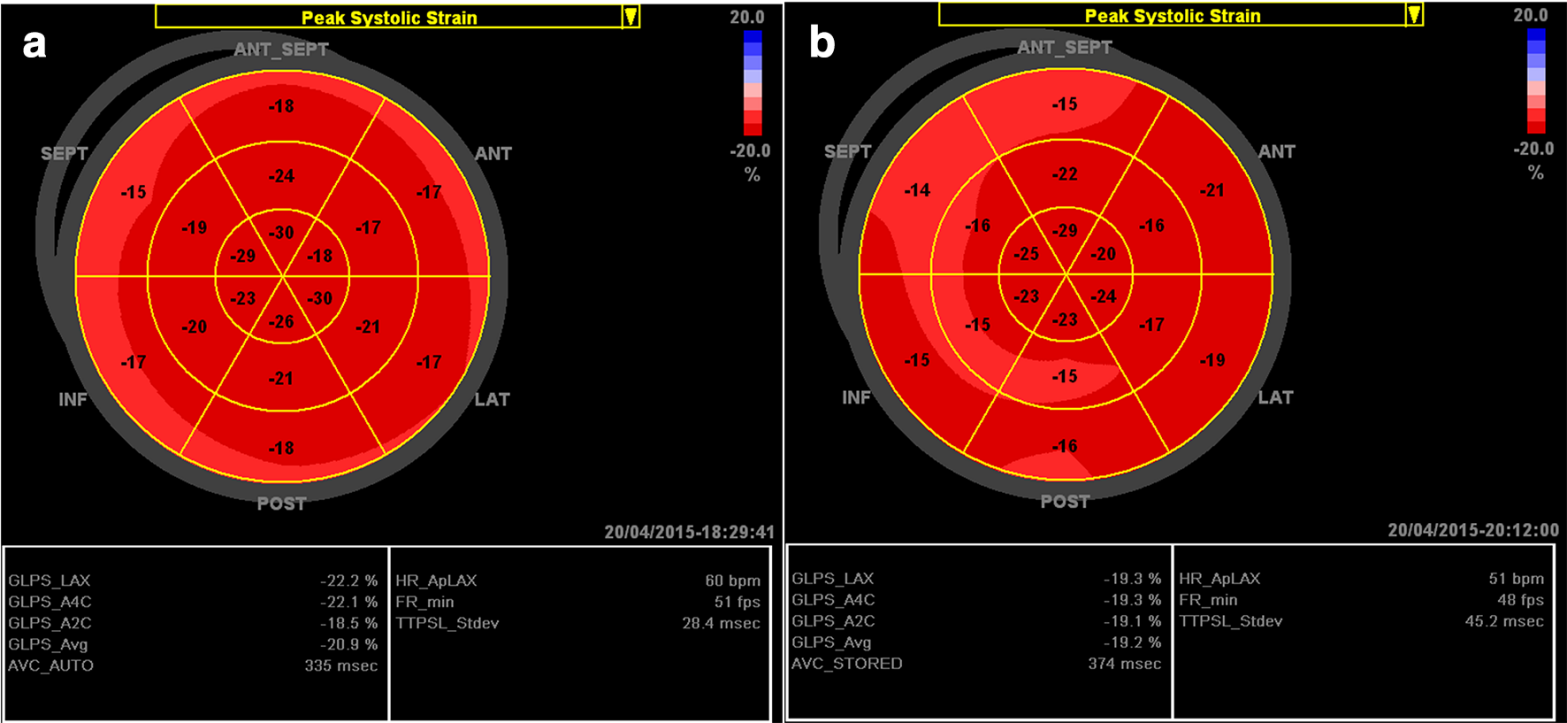
Examples of the longitudinal strain bull’s eye plot in professional basketball athletes. **a** Athlete without LVH, 30-year-old male; the end-diastolic left ventricular (LV) posterior wall thickness (LVPWd) and septal wall thickness (IVSd) are 9 mm, and LV ejection fraction (EF) is 70 %. **b** Athlete with LVH, 25-year-old male; LVPWd and IVSd are 12 mm, and LVEF is 62 %

In athletes without LVH (Fig. 2a), the apical and mid-longitudinal strains are normal (bright red) but the basal longitudinal strain is somehow lower (light red) compared to healthy subjects. The apex-basal strain gradient is therefore more pronounced in athletes without LVH compared to healthy subjects. Mildly reduced average global longitudinal strain, and much lower longitudinal strain values at the base region (pink) can be detected in athletes with physiological LVH (Fig. 2b) [16].

#### Arterial hypertension

Concentric LV hypertrophy with a wall thickness >12 mm is a typical feature in hypertensive patients [17], Some hypertensive patients still exhibit normal LV mass and wall thickness, especially at an early disease stage [18]. A localized septal thickening at the basal part (septal bulge) serves as an early echocardiographic indicator along the “hypertensive heart disease journey” [19, 20]. Most hypertensive patients with LVH also present moderately dilated aortic root and enlarged left atrium [21, 22]. LVEF usually remains normal at the early disease stage and becomes reduced in advanced stages. Diastolic dysfunction is a common echocardiographic finding in hypertensive patients, even in the absence of LVH [1, 23].

**Fig. 3:**
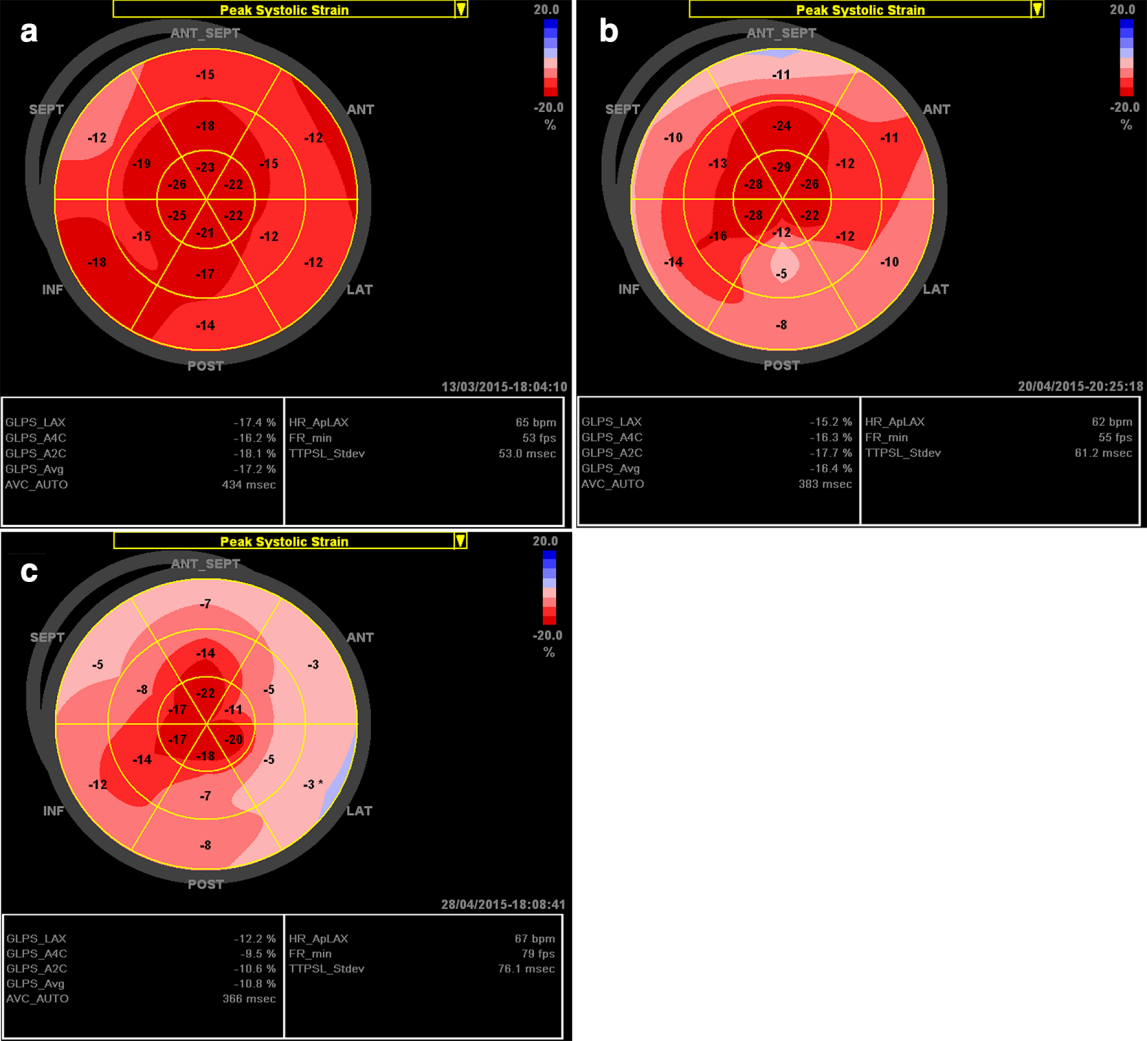
Examples of the longitudinal strain bull’s eye plot in patients with arterial hypertension. **a** Hypertensive patient with septal bulge and normal LV mass, 61-year-old female; LVPWd is 9 mm, basal septal wall thickness is 13 mm, and LVEF is 65 %. **b** Hypertensive patient with concentric LVH but normal EF, 49-year-old female; LVPWd and IVSd are 15 mm, LVEF is 75 %. **c** Hypertensive patient with concentric LVH and reduced EF, 58-year-old female, LVPWd and IVSd are 15 mm, LVEF is 48 %

The longitudinal bull’s eye plot pattern in hypertensive individuals without LVH may be very similar to that in athletes without LVH, displaying a normal average global longitudinal strain with a slightly reduced longitudinal strain at the basal segments. In hypertensive patients with septal bulge, the bull’s eye plot is characterized by a significantly reduced longitudinal strain (light red) at the basal part of the septum (Fig. 3a) [24]. In hypertensive patients with concentric LVH and normal EF, average global longitudinal strain usually remains normal or near-normal, but significantly reduced longitudinal strain patterns may be detected on multiple segments at the basal and middle levels (Fig. 3b). In cases with concentric LVH and reduced EF, reduced average global and segmental longitudinal strains are the usual findings (Fig. 3c).

The potential pathological mechanism of localized wall thickening at the basal septal segment might be associated with regional LV wall stress. Wall stress is highest at the basal septum due to the largest local radius of the LV curvature [25]. Because myocardial hypertrophy is directly related to wall stress, the basal septum usually develops a characteristic bulge. Possible reasons underlying the heterogeneous reduction in longitudinal strain might be multiple including decreased myocardial efficiency and perfusion reserve [26] and activation of signal transduction pathways related to fibrosis and apoptosis [27].

#### Hypertrophic cardiomyopathy

Idiopathic hypertrophic cardiomyopathy (HCM) is the most frequent genetically determined cardiomyopathy in adults and characterized by non-symmetric LVH in the absence of other cardiovascular or systemic diseases [28]. Typical echocardiographic findings in HCM include asymmetrical septal hypertrophy and systolic anterior motion of the mitral valve. Typically, LV end-diastolic wall thickness ≥15 mm is often observed in one or more LV myocardial segments [29], but isolated apical and other atypical distributions have also been described [30]. In cases with lesser degrees of wall thickening (13–14 mm), the diagnosis of HCM requires comprehensive evaluation on other clinical features including family history, non-cardiac symptoms and signs, electrocardiographic abnormalities, laboratory and genetic tests, and multi-modality cardiac imaging [31]. Extreme wall thickness (≥30 mm) is present in approximately 10 % of HCM patients, and has been shown to bear a particularly high risk of sudden death [32]. LV outflow tract (LVOT) obstruction owing to asymmetrical hypertrophy is found in about 25 % of cases [33]. LVOT or subaortic obstruction is defined as an instantaneous Doppler LVOT pressure gradient ≥30 mmHg at rest or during physiological provocation such as Valsalva maneuver, standing, and exercise. A gradient of ≥50 mmHg is considered to be the threshold for invasive or surgical treatment [31]. Global LV systolic function measured by EF usually remains normal or increased in most HCM patients, but regional function (particularly in segments with prominent hypertrophy) may be reduced [1].

**Fig. 4:**
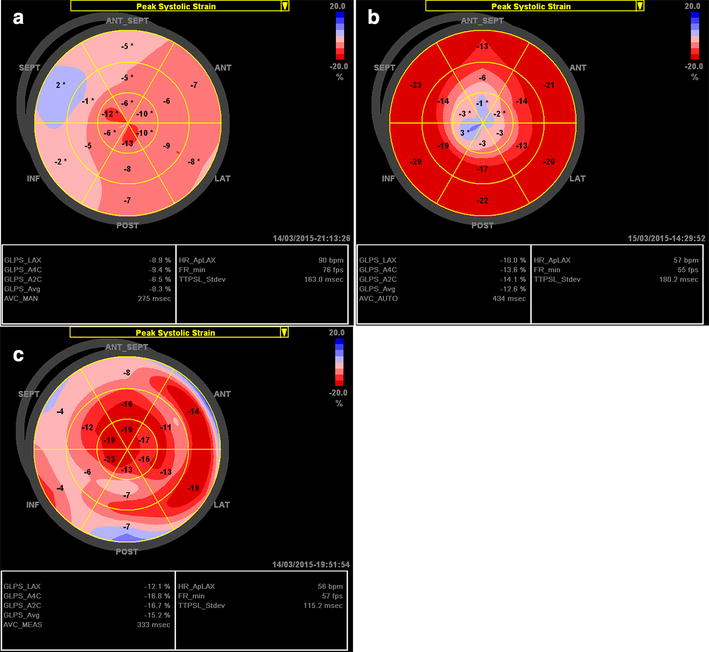
Examples of the longitudinal strain bull’s eye plot in patients with hypertrophic cardiomyopathy (HCM). **a** HCM patient with asymmetric hypertrophy, 76-year-old female; LVPWd is 12 mm and IVSd is 18 mm, LVEF is 70 %. **b** HCM patient with isolated apical hypertrophy, 59-year-old male; LVPWd and IVSd at the base are 9 mm, maximal wall thickness at the apical septum is 20 mm, and LVEF is 60 %. **c** HCM patient with concentric LVH, 70-year-old male; LVPWd and IVSd are 17 mm, LVEF is 60 %

The typical longitudinal strain bull’s eye plot pattern in HCM patients with an asymmetrical hypertrophy is characterized by a reduced average global longitudinal strain with significantly reduced strain in hypertrophic regions (Fig. 4a) [4]. In the more uncommon phenotype with isolated apical hypertrophy, the bull’s eye plot displays blue or pale pink color at the apex suggesting the absence of longitudinal deformation, surrounded by the red regions with normal strain values at the basal and middle levels (Fig. 4b).

A concentric hypertrophy occurs in about 42 % of patients with HCM [34]. This concentric form of LVH has been described to be more common in elderly HCM patients [35]. The longitudinal strain bull’s eye plot in HCM patients with concentric hypertrophy and normal EF is characterized by a mildly reduced average global and prominently reduced longitudinal strain of multiple segments (Fig. 4c).

Heterogeneous myocardial hypertrophy, disarray, and replacement fibrosis contribute to global and regional abnormalities of LV myocardial function in HCM. A recent study using 3-dimensional (3D) STI and cardiac magnetic resonance imaging (CMRI) demonstrated that global longitudinal myocardial deformation is attenuated in HCM patients, and reduced longitudinal deformation is correlated with the extent of hypertrophy. Furthermore, fibrosis detected by CMRI is presumably associated with increasing extent of hypertrophy [36]. In these severe fibrotic regions, longitudinal deformation is mostly markedly reduced with strain values lower than 5 %.

#### Amyloidosis

Amyloidosis is a multi-systemic disease characterized by the deposition of amyloid fibrils in the intercellular space of various organs [37]. Cardiac involvement, namely cardiac amyloidosis (CA), occurs in up to 50 % of patients with primary amyloidosis and indicates almost invariably a grave prognosis. Conventional echocardiographic features associated with CA include concentric left and right ventricular thickening, normal LV cavity size, dilated atria, and pericardial effusion. The myocardial texture often features a distinct “granular sparkling” [38]. Diastolic abnormalities are generally recognized as the earliest manifestation of CA [39]. LV global systolic function remains normal until the late stage of the disease [40]. With STI, CA is characterized by regional variations in longitudinal strain from base to apex. A longitudinal strain gradient with preserved systolic strain at apical segments and significantly reduced systolic strain at mid and basal segments is consistently observed [41, 42]. Previous studies have demonstrated that this pattern is specific, thus suited to differentiate patients with CA from patients with other causes of LVH [41, 43].

**Fig. 5:**
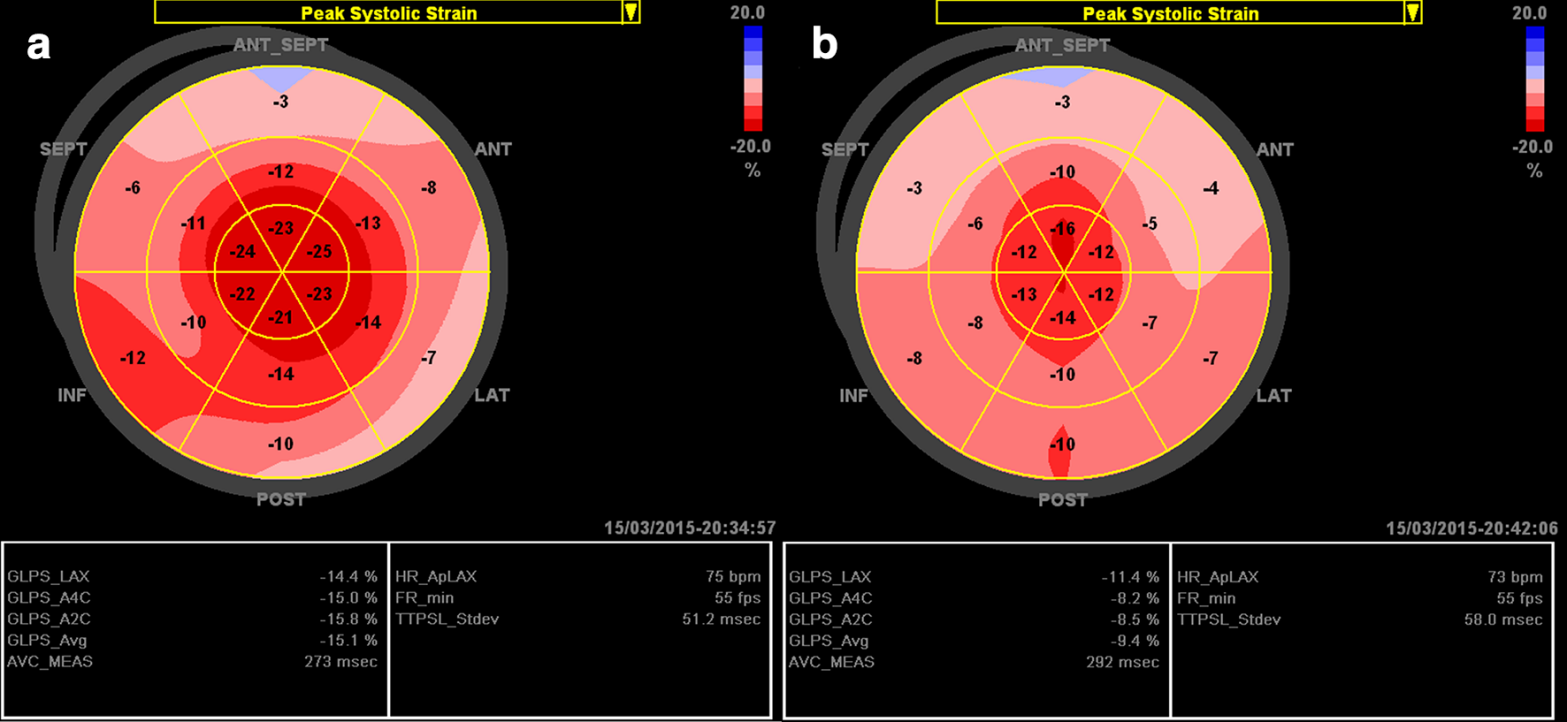
Examples of the longitudinal strain bull’s eye plot in a patient with biopsy proven cardiac amyloidosis (CA). **a** Patient with CA, 56-year-old male; LVPWd and IVSd are 13 mm, LVEF is 65 %. **b** Bull’s eye plot in the same patient 1 year later: LVPWd and IVSd are 14 mm, LVEF is 50 %

This specific relative apical sparing can be easily observed by longitudinal strain bull’s eye mapping in patients with CA. The bull’s eye plot in CA patients with normal EF shows a normal or slightly reduced average longitudinal strain, a normal longitudinal strain value at the apex of the LV (bright red), and a significantly reduced strain at all basal segments of the entire LV (pale pink to light red). Longitudinal strain at the mid regions is also reduced in some individuals (Fig. 5a). Of note, this deformation gradient is significantly higher in CA than in patients with other causes of LVH [43]. Nevertheless, with the development of disease along with a decrease in LVEF, CA patients present with a reduced average global longitudinal strain with gradually deterioration in longitudinal strain at the apex during follow-up (Fig. 5b). As a result, the base-to-apex strain gradient difference tends to become smaller in the late stage of the disease in CA.

#### Fabry disease

Fabry disease is a rare X-linked disease caused by inherited deficiency of the enzyme α galactosidase A. The lack of this enzyme leads to glycolipid storage in the myocardium associated with progressive LVH and diastolic/systolic LV dysfunction. Most patients with Fabry cardiomyopathy exhibit concentric LV hypertrophy with end-diastolic wall thickness of up to 16 mm [1, 44]. An asymmetrical phenotype could be observed in the advanced disease stage of Fabry cardiomyopathy, presenting LV concentric hypertrophy with regional wall thinning at the basal and middle posterolateral segments owing to myocardial replacement fibrosis. This replacement fibrosis can be confirmed either directly using CMRI with late enhancement (LE) or indirectly using strain rate imaging [45, 46]. In addition, a hypertrophied papillary muscle is often detected in patients with Fabry cardiomyopathy [47, 48]. Global systolic function usually remains preserved until the late stage of the disease while diastolic indices are impaired [49].

In a cross-sectional study from our group, reduced longitudinal strain was evidenced in myocardial regions exhibiting replacement fibrosis (i.e., the basal posterior and lateral LV segments) [46]. Reduced longitudinal systolic strain in the basal lateral wall was also found at very early stages of the cardiomyopathy in the absence of replacement fibrosis [44].

**Fig. 6:**
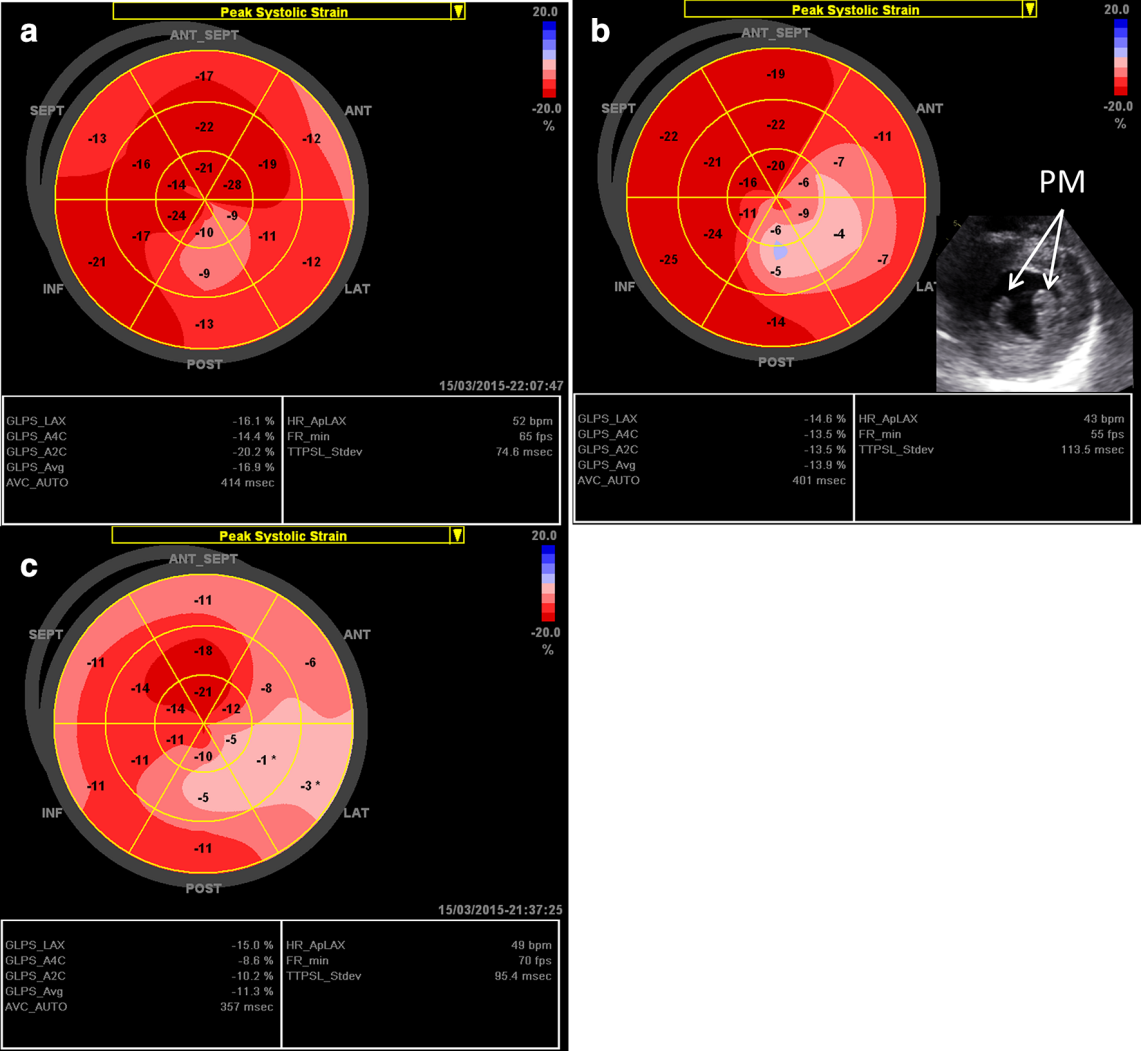
Examples of the longitudinal strain bull’s eye plot in genetically proven patients with Fabry cardiomyopathy. **a** Fabry patient with concentric LVH, 48-year-old male; LVPWd and IVSd are 13 mm, LVEF is 70 %. **b** Fabry patient with a prominent papillary muscle, 44-year-old female; LVPWd and IVSd are 14 mm, LVEF is 67 %. **c** Late-stage Fabry cardiomyopathy patient, 74-year-old female; IVSd is 18 mm, LVPWd is 13 mm, basal lateral wall thickness is 11 mm, and EF is 72 %

The usual pattern of strain bull’s eye plot in Fabry patients is a slightly reduced average global longitudinal strain despite normal LVEF (Fig. 6a) [43]. A reduced longitudinal strain at the mid segment of the lateral and posterior walls might be detected due to the presence of predominant papillary muscle (Fig. 6b). In the late stage, average global longitudinal strain is reduced and the absence of longitudinal systolic deformation (pale pink) could be detected in the basal and middle posterolateral segments with a progressive local myocardial thinning due to replacement fibrosis (Fig. 6c). Interestingly, despite similar echocardiographic morphological changes shared by Fabry patients with LVH and thinning of the posterolateral segments and HCM patients with asymmetric septal hypertrophy, the longitudinal strain bull’s eye pattern is markedly different between these two cardiomyopathies. The involved region with reduced longitudinal strain is mainly located in the septum of HCM patients, while located at the lateral and posterior walls in late-stage Fabry cardiomyopathy.

#### Friedreich’s ataxia

Friedreich’s ataxia (FA) is an autosomal recessive neurodegenerative disease caused by a guanine-adenine-adenine triplet repeat expansion in the first intron of frataxin [50]. The intronic expansion leads to a specific iron-sulfur protein (frataxin) deficiency, resulting in intra-mitochondrial iron accumulation. Besides the neurologic manifestation, cardiac involvement and endocrine involvement are also frequent [43]. A concentric LVH with an end-diastolic wall thickness of less than 15 mm is the usual echocardiographic feature [51]. Around 40 % of FA patients show concentric remodeling, 35 % show concentric hypertrophy and only 5 % display an eccentric hypertrophy [52]. Global systolic function and diastolic function remain normal in most FA patients, and only end-stage FA patients develop reduced EF with global hypokinesia and slightly dilated LV chamber [1].

**Fig. 7:**
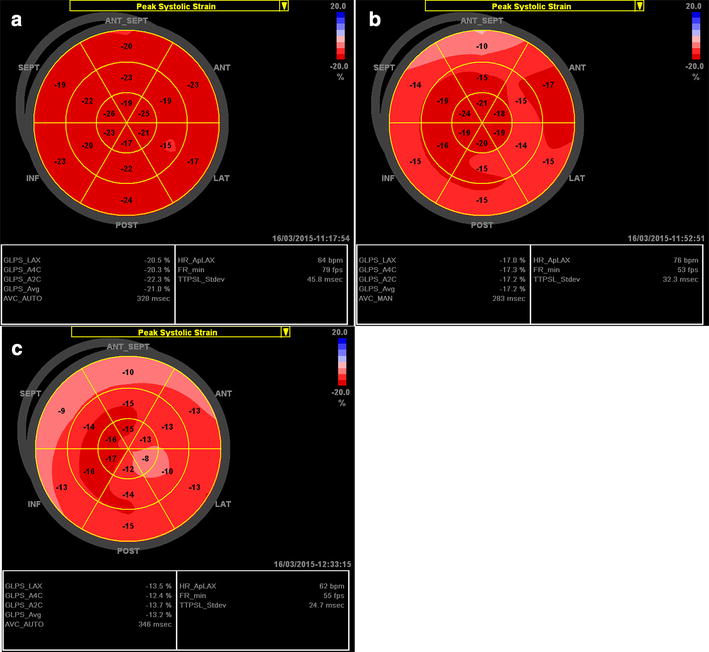
Examples of the longitudinal strain bull’s eye plot in genetically proven patients with Friedreich’s ataxia (FA) cardiomyopathy. **a** FA patient with ST-T abnormalities on ECG, 34-year-old female; LVPWd and IVSd are 9 mm, LVEF is 74 %. **b** FA patient with concentric LVH and normal EF (64 %), 21-year-old male; LVPWd and IVSd are 11 mm. **c** FA patient with concentric LVH and reduced EF (46 %), 20-year-old male; LVPWd and IVSd are 11 mm

Electrocardiographic abnormalities (ST-T changes) are often the earliest sign of FA cardiomyopathy. At this early stage, echocardiography results are usually normal and the longitudinal strain bull’s eye plot is similar pattern as healthy subjects (Fig. 7a). In FA patients with concentric LVH and normal EF, the bull’s eye plot pattern presents with a mildly reduced average global strain (Fig. 7b) [53, 54]. Myocardial fibrosis develops gradually, leading to LV wall thinning and LV dilatation during the disease progression, while EF remains preserved for a long time until the end-stage of the disease [51]. Of note, the LV wall thinning appears to be diffuse in FA cardiomyopathy, which is different from the typical findings in Fabry cardiomyopathy. The bull’s eye plot shows significantly reduced average global longitudinal strain when LVEF is reduced (Fig. 7c).

Additionally, FA cardiomyopathy shares some echocardiographic features with CA regarding morphology, including concentric LVH with a sparkling granular texture of myocardium. Different from CA, diastolic function could be normal or only mildly impaired in FA cardiomyopathy. Moreover, a longitudinal base-to-apex strain gradient, which is frequently evidenced in CA, is rarely detected in FA patient [43].

The underlying mechanisms of myocardial dysfunction in patients with FA cardiomyopathy might be associated with myocyte cellular hypertrophy, iron deposits, focal necrosis, and diffuse fibrosis [55]. CMRI with LE imaging provides evidence of fibrosis in the advanced stage of this disease, suggesting that fibrosis might be associated with subsequent myocardial dysfunction [51].

### Technical limitations

The major technical limitation for longitudinal strain bull’s eye acquisition is the need of high-quality echocardiographic images in standard apical views. Significant risk of misdiagnose presents when analyzing patients with unsatisfactory imaging. In our experience, 2–3 h intensive training is enough to get satisfactory analysis in case of optimal imaging. However, expert know-how is essential to deal with unsatisfactory imaging.

Furthermore, similar heart rate and suitable frame rate in all three apical views are essential prerequisites to configure the bull’s eye plot. Thus, the application of this diagnostic tool based on 2D STI remains limited in patients with arrhythmia. 3D echocardiography with tri-plane mode image acquisition allows a more accurate evaluation in myocardial deformation via imaging of different apical views from the same heart cycle simultaneously with sufficient temporal and spatial resolution [56]. Although tri-plane mode with 3D echocardiography improves the different heart rate issues, the need for high imaging quality remains a challenge even in the era of 3D echocardiography for bull’s eye acquisition.

Additional limitations of STI include smoothing, frame rate dependency, and curvature dependency [46].

STI-derived stain and strain rate curves rely on both spatial and temporal smoothing, and a spline smoothing function enables smoother curves. However, excessive smoothing may lead to undersampling. The regularization of spatial and temporal smoothing function is available in the most commercial applications. Segmental strain values could be affected while adjusting the smoothing parameters [57]. Thus, it is recommended that smoothing should be limited to the necessary minimum in deformation analysis [7]. Reverberations are sometimes tracked or interfere with the frame-by-frame tracking, which might result in drift or incorrect calculation of myocardial deformation.

### Reproducibility of longitudinal strain bull’s eye plot

The bull’s eye plot serves as a reconstructional modality based on global and regional longitudinal systolic strain measurements. Its reproducibility therefore is consistent with the reproducibility of speckle tracking derived longitudinal strain measurements, which have been well demonstrated in a number of studies. In general, accuracy of 2D speckle tracking-derived strain measurements by the current available software was acceptable with high intra-vendor reproducibility [coefficients of variation (CV) <5 %] in clinical models including normal, LV hypertrophy, dilated, and exercise model [58]. A recent report from a large and epidemiologic community-based study demonstrated an excellent reproducibility of global longitudinal strain with a CV ≤4 % [59]. However, the variability of segmental strain values was somehow higher compared to global strain values [60]. Segmental analysis showed that inter- and intra-observer reproducibility of longitudinal strain measurements was better at middle segments (inter- and intra-observer CV 6.3 ± 3.5 and 6.0 ± 3.2 %) than at basal and apical segments (inter- and intra-observer CV 8.5 ± 5.6 and 8.1 ± 5.2 % for basal segments, 9.0 ± 5.2 and 11.0 ± 6.3 % for apical segments) [59].

### Clinical implication

The bull’s eye plot offers an intuitive visual overview of the global and regional LV myocardial function status in various cardiomyopathies with LVH. The bull’s eye longitudinal strain mapping is clinically feasible and the plot patterns derived by a further expansion of this technique in clinical practice provide clues to the etiology of cardiomyopathies.

Three information can be extracted from 2D STI. First, the investigator gets information of the overall LV function by averaged strain. Global longitudinal deformation is closely associated with the severity of LVH and LVEF. A significant concentric LVH with end-diastolic wall thickness of more than 16 mm already shows reduced average global strain in the bull’s eye plot even in case of preserved LVEF. Average global longitudinal systolic strain is reduced along with reduced LVEF in all types of cardiomyopathy.

Second, a disease-related typical deformation pattern can be detected and the bull’s eye map could provide a valuable clue for the final diagnosis in some patients with unclear LV hypertrophy. It is a common sense that the athlete’s heart is associated with physiological hypertrophy, often presents with a normal strain pattern. Nevertheless, the bull’s eye mapping unexpectedly demonstrates mildly reduced longitudinal strain at the basal segments in some athletes even in the absence of LVH. The significance of this finding warrants future studies to explore the potential clinical relevance of this strain change. The isolated septal bulge with localized longitudinal strain abnormality serves as an early sign in hypertensive patients. The bull’s eye plot pattern in HCM patients is closely associated with the location and severity of myocardial hypertrophy. Of note, Fabry patients at the advanced stage sometimes also exhibit an asymmetric hypertrophy with thick septum and thin lateral and posterior walls due to replacement fibrosis. In contrast to HCM, significantly reduced longitudinal strain in the bull’s eye plot is detected at the lateral and posterior walls in Fabry cardiomyopathy but not that significant in hypertrophied septum. Additionally, a significantly reduced deformation at the middle lateral and posterior walls is frequently observed in Fabry patients due to hypertrophied papillary muscle. Pronounced longitudinal base-to-apex strain gradient serves as the distinct feature in CA patients. The bull’s eye pattern in FA cardiomyopathy appears to be nonspecific and diastolic function evaluation could aid to differentiate FA cardiomyopathy from CA.

Third, in patients where the diagnosis is known, the combination of averaged strain together with the bull’s eye pattern allows staging on the severity of cardiac involvement.

In our view, the bull’ eye display provides more direct viewing and physicians could easily make direct impression on what he/she see, in one word, dealing more directly by viewing a picture rather than reading the numbers of the classical strain. However, the bull’s eye display is obtained based on the classical strain measurements. This display could not replace the classical strain but provides more a merit to know the strain distribution pattern by directly viewing the bull’s eye picture. Automatic computer-aided diagnosis and statistical analysis techniques might help and provide fast bull’s eye display for patients with good imaging conditions. Again, these techniques will face similar difficulties as manual Bull’s eye display when analyzing patients with bad imaging. In the future, computer-based “pattern recognition” might help detect disease-related deformation patterns for all the different hypertrophic cardiomyopathies.

## Conclusions

In conclusion, although the bull’s eye plot could provide additional important information in patients with cardiomyopathies with LVH, a comprehensive cardiac workup remains essential to confirm the diagnosis of cardiomyopathies, including the evaluation of family and clinical history, non-cardiac involvements assessment, laboratory and eventually genetic tests, ECG, and multi-modality cardiac imaging (echocardiography, CMRI).

## Abbreviations

LVH: left ventricular hypertrophy
STI: speckle tracking imaging
AFI: automated function imaging
LV: left ventricular
2D: 2-dimensional
ROI: region of interest
EF: ejection fraction
HCM: hypertrophic cardiomyopathy
LVOT: LV outflow tract
3D: 3-dimensional
CMRI: cardiac magnetic resonance imaging
CA: cardiac amyloidosis
FA: Friedreich’s ataxia
